# Effect of plant nitrogen and water status on the foraging behavior and fitness of an omnivorous arthropod

**DOI:** 10.1002/ece3.1788

**Published:** 2015-11-04

**Authors:** Peng Han, Yongcheng Dong, Anne‐Violette Lavoir, Stéphane Adamowicz, Philippe Bearez, Eric Wajnberg, Nicolas Desneux

**Affiliations:** ^1^ INRA (French National Institute for Agricultural Research)Univ. Nice Sophia AntipolisCNRSUMR 1355‐7254Institut Sophia Agrobiotech06903Sophia AntipolisFrance; ^2^ Hubei Insect Resources Utilization and Sustainable Pest Management Key LaboratoryCollege of Plant Science and TechnologyHuazhong Agricultural UniversityWuhan430070China; ^3^ INRAUR1115 PSH228 Route de l'aérodrome84914AvignonFrance

**Keywords:** Bottom‐up effect, fecundity, feeding behavior, longevity, omnivorous predator, plant preference

## Abstract

Omnivorous arthropods make dietary choices according to the environment in which they forage, mainly availability/quality of plant and/or prey resources. Such decisions and their subsequent impacts on life‐history traits may be affected by the availability of nutrients and water to plants, that is, through bottom‐up forces. By setting up arenas for feeding behavior observation as well as glasshouse cages for plant preference assessment, we studied effects of the presence of prey (Lepidoptera eggs) and nitrogen/water availability to host tomato plants on the foraging behavior and life‐history traits in the omnivorous predator *Macrolophus pygmaeus* (Heteroptera: Miridae). In the absence of prey, the predator fed equally on the plants treated with various levels of nitrogen and water. In the presence of prey, however, the feeding rate on plants decreased when the plant received low water input. The feeding rate on prey was positively correlated with feeding rate on plants; that is, prey feeding increased with plant feeding when the plants received high water input. Moreover, plants receiving high water input attracted more *M. pygmaeus* adults compared with those receiving low water input. For *M. pygmaeus* fitness, the presence of prey enhanced its fertility and longevity, but the longevity decreased when plants received low compared with high water input. In conclusion, the omnivorous predator may be obliged to feed on plants to obtain water, and plant water status may be a limiting factor for the foraging behavior and fitness of the omnivorous predator.

## Introduction

Omnivores, that is, organisms feeding on two or more trophic levels, are widespread across many animal taxa. Such a feeding habit may partly explain the fact that food webs are highly interconnected and complex. Omnivory has been thought to play important roles in shaping the natural community structure and dynamics (Naranjo and Gibson 1996; McCann et al. 1998; Coll and Guershon 2002; Eubanks 2005; Thompson et al. 2007; Chubaty et al. 2014), and specifically the arthropod communities (McMurtry and Croft 1997; Coll 1998; Eubanks and Denno 2000; Symondson et al. 2002; Eubanks et al. 2003). Foraging has been considered one of the most important aspects in behavioral ecology study in omnivorous arthropods. One reason could be that foraging and in turn their efficacy as predators (hereafter named omnivorous predators) is strongly dependent on the environment in which they forage (Eubanks and Denno 1999, 2000).

Omnivorous predators need to acquire nutrients from both their host plants and prey (Coll and Guershon 2002; Lundgren 2009). Their dietary choice, for example, feeding on plants or prey, may depend on their habitat characteristics, particularly the availability and density of prey (Agrawal et al. 1999; Montserrat et al. 2004; Gillespie et al. 2012), the plant quality (Eubanks and Denno 1999, 2000; Janssen et al. 2003; Gillespie et al. 2012), as well as the external abiotic factors such as water availability (Gillespie and McGregor 2000; Sinia et al. 2004). A theoretical framework with three contrasting hypotheses has been proposed to describe the main relationships between plant and prey feeding activities of omnivorous predators (Gillespie and McGregor 2000): (1) *Switching hypothesis* – the omnivorous predators switch between plant and prey foods, for example, the prey feeding rate increases as plant feeding rate decreases when the plant quality becomes low; (2) *Facilitation hypothesis* – plants contain key components (nutrients or water) that may facilitate prey feeding through enhanced digestion or assimilation and the prey feeding rate thus increases with plant feeding rate; and (3) *Independence hypothesis* – the prey and plant feeding rates are independent. Most studies have supported the *switching hypothesis* that omnivores tend to consume more prey when plant quality becomes low, that is, lower palatability and higher plant defense level (Coll 1996; Agrawal et al. 1999; Eubanks and Denno 1999, 2000; Janssen et al. 2003; Eubanks 2005; Kaplan and Thaler 2011). Notably, a more recent study by Gillespie et al. (2012) has emphasized the importance of plant context in determining the foraging of omnivorous predators and their role in food web. However, it remains elusive how omnivorous predator's foraging behavior varies with plant quality change due to different resources input, even though several studies have assessed the effects of water and nutrients applied to host plants on the fitness of some omnivorous bugs (Groenteman et al. 2006; Seagraves et al. 2011).

Plant quality can be manipulated by various biotic or abiotic factors. Among the abiotic factors, water availability to plants can influence plant–insect interactions by changing the nutritional value of plant food (Schoonhoven et al. 2005), as well as the plant resistance to insect herbivory (Gutbrodt et al. 2011). Nitrogen, one of the most crucial nutrients for plants, has been considered another important factor influencing the phytophagy in insects (Mattson 1980; Schoonhoven et al. 2005). Since omnivorous predators are commonly used in biological control programs (Symondson et al. 2002) in which nitrogen and water can be readily manipulated in given contexts (e.g., glasshouse conditions), it is of great importance to understand how nitrogen and water inputs to plants could affect their foraging behavior and fitness.

In the present study, we set up a full factorial combination design to examine the combined effects of nitrogen (high vs. low) and water (high vs. low) input, together with prey availability (presence vs. absence), on (1) the foraging behavior, and (2) key life‐history traits, that is, fertility and longevity of an omnivorous predator. We hypothesize that plants subjected to varying nitrogen and water inputs may trigger bottom‐up effects on its foraging behavior as well as fitness. To test our hypothesis, we set up a tri‐trophic system “tomato–prey eggs–omnivorous predator” to carry out a series of bioassays under laboratory and glasshouse conditions. Although many species belonging to Heteroptera (true bugs) are strictly phytophagous and are known as serious agricultural pests (Tan et al. 2012; Haye et al. 2014; Pan et al. 2014; Tillman 2014), omnivory is quite common in many other species in this taxa (Cohen 2000). The omnivorous predator *Macrolophus pygmaeus* Rambur (Heteroptera: Miridae) is used in the present study. Its phytophagy has been characterized by feeding on plant materials (e.g., leaves and stems) and plant product (e.g., pollen and nectar) (Castañé et al. 2011). The species also attacks various arthropods, for example, whiteflies, thrips, aphids, and lepidopteran pests (Margaritopoulos et al. 2003; Biondi et al. 2013; Bompard et al. 2013; Chailleux et al. 2013a,b; Zappalà et al. 2013; Velasco‐Hernández et al. 2015). The case study on *M. pygmaeus* may not only help to gain a better knowledge of foraging behavior of omnivorous predators, but also help to guide the optimized use of omnivorous predators in biological control programs.

## Materials and Methods

### Biological materials

**Figure 1:**
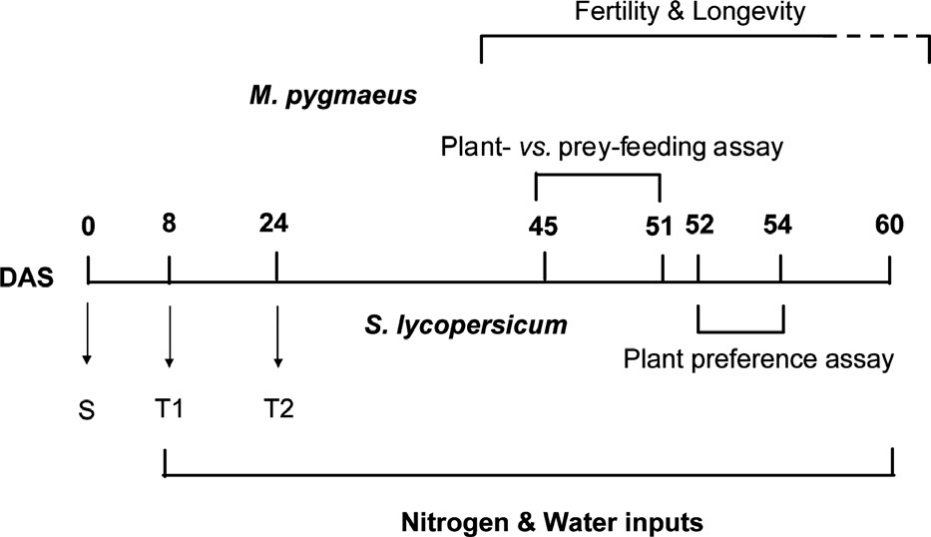
Calendar in days of plant cultivation, nutritional treatment, and all the assays of *Macrolophus pygmaeus* throughout the plant developmental stage; S: plant sowing; T1/T2: routine plant transfer; DAS: days after sowing.

Tomato plants (*S. lycopersicum* L. cv. Marmande) were grown from seeds in a climatic chamber (L:D 12:12, 24 ± 1°C, 65 ± 5% RH) as reported by Han et al. (2014). Germination was carried out in small plastic pots (7 × 7 × 6.5 cm, TEKU, Rixheim, France) filled with compost (Tonusol, Draguignan, France). In order to control nutrition, after rinsing the roots, the plantlets were transferred to pots containing an inert substrate (Perlite Italiana srl, Corsico, Italy) the first time (T1) at 8 days after sowing (DAS; Fig. 1) and the second time (T2) to larger pots (diam. 10 cm, height 9 cm) at 24 DAS. The *M. pygmaeus* colony was reared in cages placed in environmental chambers (L:D 16:8, 25 ± 1°C, 70 ± 10% RH). It was fed UV‐irradiated *Ephestia kuehniella* eggs (Pyralidae: Lepidoptera) and pollen (Famille MICHAUD Apiculteurs, France), and tomato plants were provided as oviposition sites. The eggs were provided by Biotop (Livron‐sur‐Drôme, France) and were stored at 4°C. The colony was initiated 2 years prior to the start of the experiments using adults collected from tomato glasshouses in south France, and new *M. pygmaeus* were added twice a year. All tests were conducted using 7‐day‐old mated *M. pygmaeus* adult females. They were isolated individually in glass vials with a piece of tomato stem 24 h before the experiments.

### Plant nutrition: nitrogen and water inputs

Nutritional treatment design was adapted from Han et al. (2014). From 8 DAS onwards, a full nutrient solution adjusted to 5.5 pH was supplied daily to plants in a fully crossed design combining two nitrogen input levels (HN vs. LN, for high vs. low nitrogen, respectively) with two water input levels (HW vs. LW, for high water vs. low water, respectively). Each treatment was applied to 33 plants, 132 plants in total.

**Figure 2:**
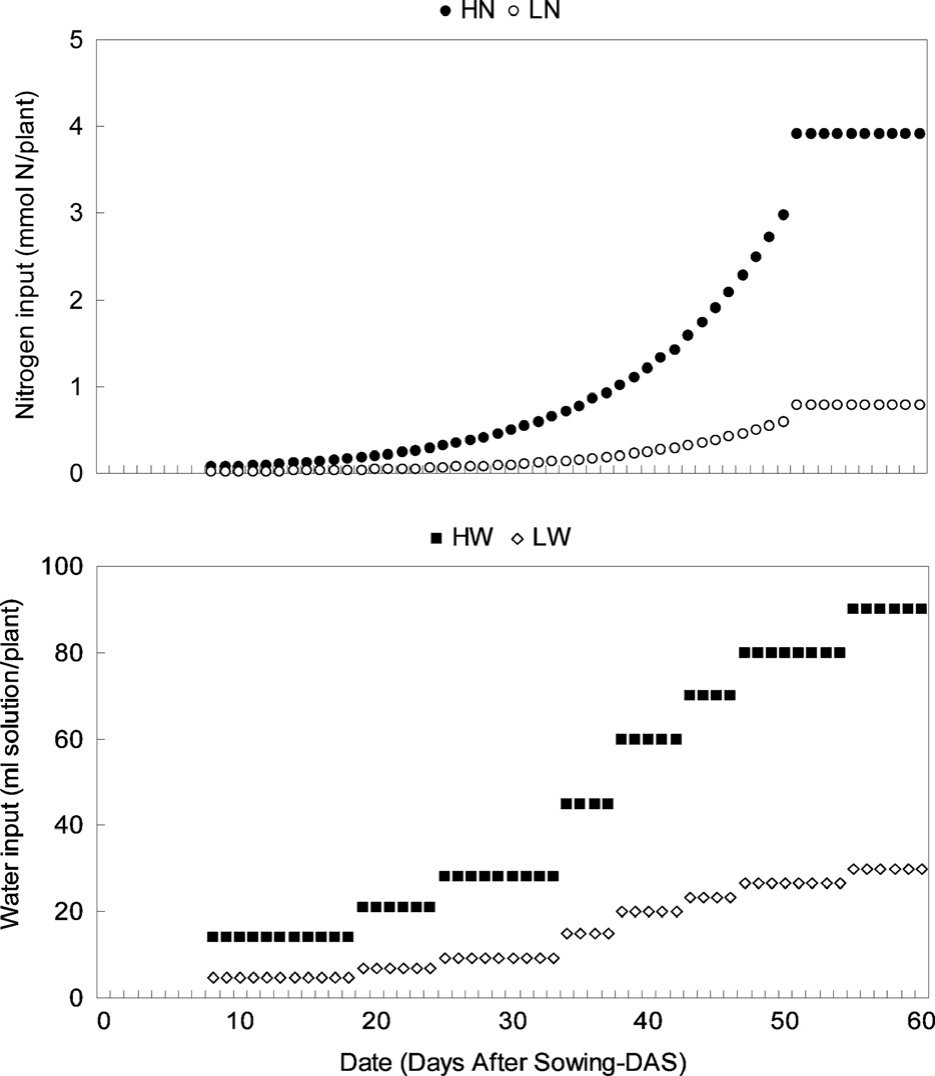
Nutritional treatments applied to the tomato plants during 60 days of growth: amount of daily nitrogen input (mmol) and water volume input (mL of solution) per plant throughout the growth period; DAS, days after sowing; HN, high nitrogen input; LN, low nitrogen input; HW, high water input; LW, low water input.

The amounts of nutrient inputs and volumes of feeding solution supplied to plants increased daily depending on the plant development stage. Indeed, the amount of nitrogen taken by plants is known to be controlled by the increase in their daily dry mass (Le Bot et al. 1998). Based on the knowledge of the tomato growth curve previously measured under our laboratory conditions, we therefore calculated the daily amounts of nitrogen required to produce optimal dry mass during approximately 52 days of growth (namely V_h,_ i.e., “high nitrogen” treatment). We used three stock solutions to provide nitrate, phosphate, and sulfate salts independently (+ Fe and micronutrients). The following concentrations were used (NO_3_: 1 mol/L H_2_PO_4_: 0.21 mol/L; SO_4_: 0.055 mol/L; K: 0.641 mol/L; Ca: 0.215 mol/L; Mg M: 0.114 mol/L). Kanieltra trace elements (Hydro Azote, France) were given as well as formula 6 Fe and EDTA‐Fe at the following concentrations (in *μ*mol/L in the SO_4_^2−^ stock solution: Mo: 20; Mn: 815; Zn: 227; Cu: 33; B: 1444; Fe: 3760). To differentiate N inputs, two different doses, namely V_h_ (high nitrogen: HN) and V_h_/5 (low nitrogen: LN) of the nitrate stock solution, were used to fertilize the plants on a daily basis. These doses were added to the water intake (see below) by each plant in order to set the high and low nitrogen inputs, respectively (Fig. 2) on a daily basis following the same increase pattern as tomato growth curve. We applied a “step increase” pattern to daily water inputs (Fig. 2). High water input (HW) was determined empirically as the amount capable of fully saturating the perlite substrate without visible drainage, that is, field capacity. One‐third of such water volume was applied daily to set the low water input treatment (LW).

### Plant‐ versus prey feeding assay

The assay was carried out under laboratory conditions (L: D 16: 8, 24 ± 1°C, 65 ± 5% RH) in INRA Sophia‐Antipolis, France, in March 2014. The observation of *M. pygmaeus* foraging behavior was conducted by confining them individually on a tomato leaflet trapped within an arena, that is, 10‐cm Petri dishes with a 7‐cm ventilation hole covered by fine nylon mesh screen. Adult females were individually released into the arena and experienced a 48 h‐period (to get used to the arena conditions) without prey available before foraging behavior observations. Two prey treatments were tested: (1) the presence (prey consisted in 100 *E. kuehniella* eggs, provided daily) or (2) absence of prey. Therefore, the whole experimental setup was designed as 8 treatments (2 × 2 × 2 factorial designs): HN versus LN, HW versus LW, and prey presence versus absence. There were 33 replicates (arenas) from each treatment combination with one insect confined in each arena. From 45 to 51 DAS, direct observations using common hand‐held magnifier were performed twice daily (10:00–11:00 a.m. and 3:00–4:00 p.m.) (Fig. 1). During these observations, each *M. pygmaeus* was initially observed for 10 sec to check and locate it in the arena, then the behavior was observed during the following 15‐sec interval and recorded as: (1) plant feeding, (2) prey feeding, (3) walking, or (4) resting or grooming (Desneux and O'Neil 2008). The stylets inserting into leaf tissues over 6s was recorded as “plant feeding”. We judged the plant feeding behavior based on this time range since we found *M. pygmaeus* often spends 6 ± 0.8 sec on inserting their stylets into the leaf tissue and drawing out without duration of halt in the preliminary assay. Stylets inserting into prey eggs with at least a short duration of halt was recorded as “prey feeding”. The “resting” behavior mainly involves grooming of stylets or remaining still. The “walking” behavior involves searching for food (e.g., plant feeding site, prey items or other important resources), or for a suitable site for oviposition, etc. Each individual was observed twice per day and the total number of observation events ranged from 589 to 672 for each treatment mainly because a few individuals died during the course of the experiments. The plant feeding rate was calculated as the ratio of plant feeding behavior observations divided by total number of all observed behaviors (i.e., feeding activities, walking, and resting). In the same way, the prey feeding rate was calculated as the ratio of prey feeding behavior observations divided by total number of all observed behaviors.

### Predator fertility and longevity

After the feeding observation, a continued supply of *E. kuehniella* eggs was available (10–20 eggs daily) to each predator. Emerging nymphs within the arenas were counted and removed daily in order to estimate the fertility of *M. pygmaeus* females. To estimate longevity, the predator was considered dead when it remained completely immobile.

### Plant preference assay under glass conditions

The assay was conducted in one of the glasshouse compartments in INRA Sophia‐Antipolis, France, in March 2014. The assay was carried out in a mesh cage (length × width × height: 2 × 1 × 1 m) under glasshouse conditions (27 ± 3°C, 65 ± 10% RH). About 80 *M. pygmaeus* adults (sex ratio nearly 1:1) were sampled as one batch from the *M. pygmaeus* colony and then supplied with *E. kuehniella* eggs only within glass vials without water from 8:00 a.m. to 3:00 p.m. Three to four batches were prepared for each day. Between 3:00 p.m. and 5:00 p.m., adults were released into a cage containing plants treated with various nitrogen and water inputs. In each trial (replicate), four plants were used with one plant from each treatment (2 × 2 factorial designs) as specified earlier: two levels of nitrogen by two levels of water input. The plants were randomly arranged in a circle, and the tested adults were released in the center of the cage. Adults were given 5 min to choose their plant habitat. In a pilot experiment, we found that some *M. pygmaeus* individuals moved frequently among different plants during the first 5 min. We assume that they need to take an average of 5 min to choose a suitable plant until they settle down. The pilot experiment was carried out using the same design under the same conditions mentioned above. Thereafter, the number of adults found on each plant was recorded. Ten trials (replicates) were performed. The experiment was conducted over a 3‐day period (52–54 DAS).

### Data analysis

Shapiro and Bartlett tests were used to assess variance homogeneity and normality of all the data, respectively. Independent factorial two‐way ANOVAs (nitrogen × water) were performed to test the effects of nitrogen and water on (1) plant and prey feeding rates of *M. pygmaeus* in the presence of prey, (2) plant feeding rate of *M. pygmaeus* in the absence of prey, and (3) *M. pygmaeus* plant preference. We did not consider “date” as an independent factor in the analyses of the predator plant preference data since this experiment only lasted for 3 days and all the assays were repeated during exactly the same time period of the day. In addition, linear regression analysis was conducted on plant versus prey feeding rates to test the relationship between the two feeding activities. Factorial three‐way ANOVAs (nitrogen × water × prey) were performed on *M. pygmaeus* fertility and longevity datasets. Multiple comparisons assessing the effect of nitrogen and water input on *M. pygmaeus* plant feeding rate (prey absence), as well as on plant preference, were based on Tukey's post hoc tests. Similar multiple comparisons were carried out to assess the effect of nitrogen, water input, and prey availability on *M. pygmaeus* fertility and longevity. All statistical analyses were done using R software.

## Results

### Omnivorous predator feeding behavior

**Figure 3:**
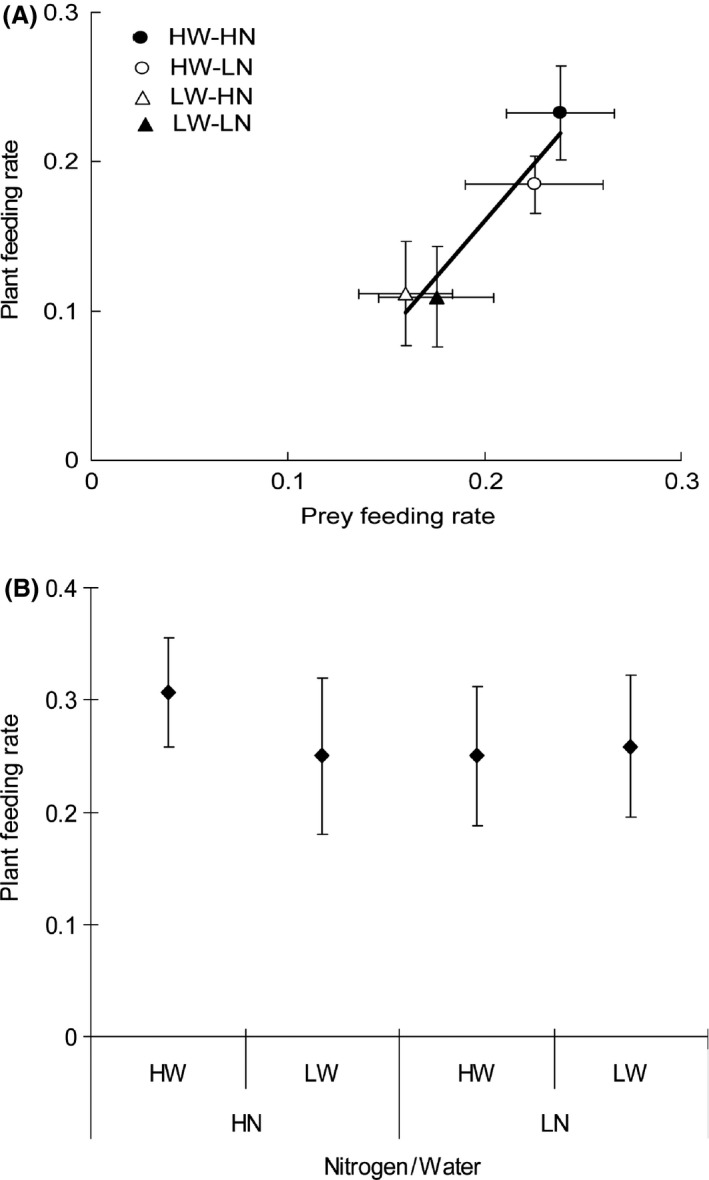
Predator plant‐ versus prey feeding behavior observation in the arenas: (A) Relationship between average plant feeding and prey feeding rate (±SE, the solid line is a linear regression), and (B) average plant feeding rate (±SE), when plants were treated with high and low nitrogen inputs (HN and LN) in combination with high and low water inputs (HW and LW) in the absence of prey.

In the presence of prey, plant feeding rate increased with prey feeding rate when higher water input was supplied (*R* ^2^ = 0.935, *P* = 0.034; Fig. 3A). However, both feeding rates were not affected by nitrogen input (Table 1). Irrespective of nitrogen treatment, the average plant and prey feeding rate were 0.21 ± 0.02 and 0.23 ± 0.02 on the plants receiving high water input, whereas they were 0.11 ± 0.02 and 0.17 ± 0.02 on those treated with low water input. By contrast, in the absence of prey, the plant feeding rate was neither affected by nitrogen nor by water input (Table 1). The average rate was 0.27 ± 0.03 (Fig. 3B).

**Table 1:** Effects of host plant nitrogen input (high vs. low) and water input (high vs. low) on feeding behavior of *M. pygmaeus* in the presence or absence of prey. Factorial ANOVAs were performed. Bold text indicates significant effects

Plant‐ versus prey feeding assay in microcosm	Presence of prey				Absence of prey	
	Plant feeding rate		Prey feeding rate		Plant feeding rate	
Source of variation	*F*_1,16_	*P* values	*F*_1,16_	*P* values	*F*_1,16_	*P* values
Nitrogen	0.64	0.43	0.00	0.96	0.15	0.69
Water	**10.50**	**0.00**	**4.87**	**0.04**	0.15	0.70
Nitrogen × water	0.53	0.47	0.25	0.62	0.27	0.60

### Omnivorous predator plant preference assay

**Figure 4:**
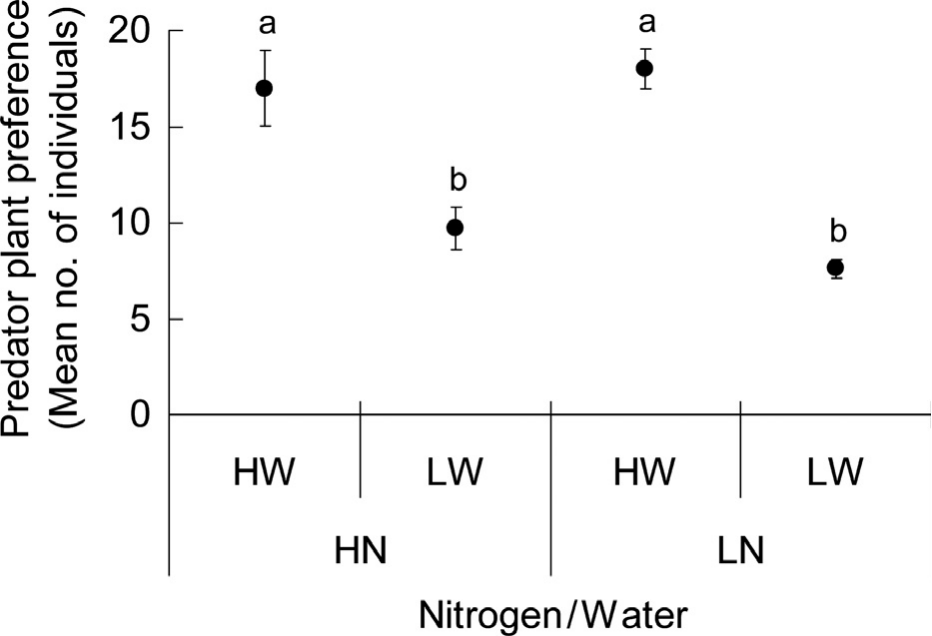
Plant preference (mean no. of individual ±SE) by *Macrolophus pygmaeus* adults when the plants were treated with high and low nitrogen inputs (HN and LN) in combination with high and low water inputs (HW and LW). Values followed by the same letters are not significantly different (*P *>* *0.05, ANOVA followed by Tukey's post hoc tests).

Plants receiving low water inputs attracted on average 50. 6% fewer predator adults compared to high water input (Fig. 4: *F*_3, 36_ = 17.23, *P *<* *0.001). However, the predator preference was not affected by the nitrogen input (Table 2).

**Table 2:** Effects of host plant nitrogen input (high vs. low), water input (high vs. low), prey food (presence vs. absence) and their interactions on plant preference, fertility, and longevity of *M. pygmaeus*. Factorial ANOVAs were performed. Bold text indicates significant effects. The factor “prey” was not tested during the plant preference assay since all the predator groups were provided with prey food in this case

Biological traits	Plant preference		Fertility		Longevity	
Source of variation	*F*_1, 36_	*P* values	*F*_1,152_	*P* values	*F*_1,152_	*P* values
Nitrogen	0.19	0.66	0.84	0.36	0.42	0.51
Water	**49.95**	**<0.001**	0.01	0.91	**5.91**	**0.01**
Prey	–	–	**112.0**	**<0.001**	**227.3**	**<0.001**
Water × nitrogen	1.53	0.22	0.37	0.54	0.39	0.53
Water × prey	–	–	0.34	0.56	**9.42**	**0.003**
Nitrogen × prey	–	–	2.53	0.11	0.39	0.52

### Omnivorous predator fertility and longevity

**Figure 5:**
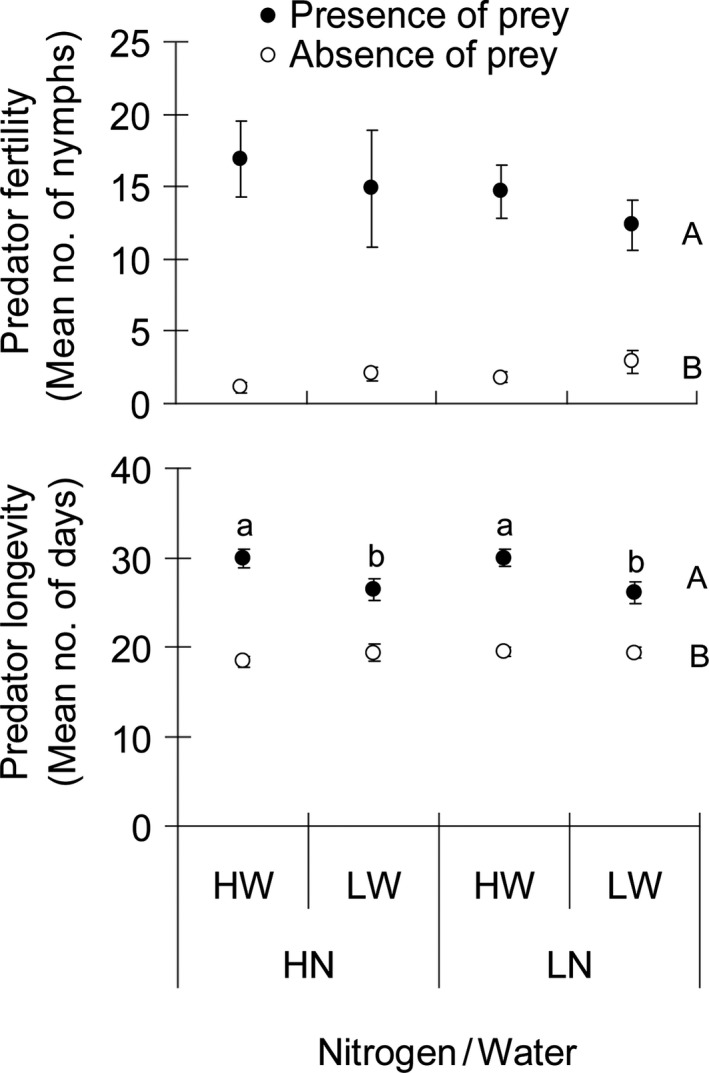
Effects of nitrogen and water inputs and presence of prey on average (±SE) fertility (no. of nymphs) and average (±SE) longevity (days) of *Macrolophus pygmaeus*. The plants were treated with high and low nitrogen inputs (HN and LN) in combination with high and low water inputs (HW and LW). Values followed by the same letters are not significantly different (*P *>* *0.05, ANOVA followed by Tukey's post hoc test). Capital letters (A, B) indicate the comparison between “Presence of prey” and “Absence of prey”. Small letters (a, b) indicate the comparison among the nitrogen and water treatments (No letter is present when the differences are not significant).

The predator exhibited much lower fertility in the absence of prey compared with the presence of prey. However, fertility was independent of nitrogen and water treatments at both prey treatments (Table 2; Fig. 5). A significant interaction between water and prey was found on predator longevity (Table 2). The negative effect of low water input on predator longevity was significant only in the presence of prey (Fig. 5: “presence of prey”: *F*_3,76_ = 3.469, *P *=* *0.020; “absence of prey”: *F*_3,76_ = 0.739, *P *=* *0.532).

## Discussion

Our data demonstrated that plants subjected to varying nitrogen and water inputs may trigger bottom‐up effects on *M. pygmaeus* foraging behavior as well as its fitness. In the absence of prey*, M. pygmaeus* fed equally on the plants subjected to various nitrogen and water inputs. However, when prey was present, there was a positive correlation between plant and prey feeding activities by *M. pygmaeus*; that is, the plant feeding increased with the prey feeding when plants received more water input. In addition, we demonstrated that (1) availability of prey increased *M. pygmaeus* fertility and longevity, and (2) water limitation led to decreased *M. pygmaeus* longevity when prey was available.

The omnivorous predator enhanced both plant and prey feeding activities when the plants received sufficient water input (Fig. 3A). The positive correlation between the two feeding activities may indicate a physiological link between the two. Water may be a crucial factor responsible for such correlation since plant feeding is a means of acquiring water for the omnivorous predator (Gillespie and McGregor 2000; Sinia et al. 2004). Indeed, plant material may provide essential nutrients to help carnivory (Sinia et al. 2004; Eubanks and Styrsky 2005). However, we lack the data for identification and quantification of plant compounds that could be linked to prey digestion. At least, this highlights the importance of using both plant and prey diets in terrestrial omnivorous arthropods (Coll and Guershon 2002).

Water proves to be a critical factor for foraging of *M. pygmaeus*. High water input to plants greatly improved both types of feeding activities in *M. pygmaeus* (Fig. 3A). This was consistent with the findings by Sinia et al. (2004) showing that water acquired through plant feeding was required for prey predation/digestion. Furthermore, our behavioral assays showed that plants receiving high water input attracted more *M. pygmaeus* adults compared with those receiving low water input (Fig. 4), thus providing further evidence on the importance of water availability for the predator. We could not avoid the possibility of substrate water choice by *M. pygmaeus*. However, such effect is considered minor since most testing *M. pygmaeus* have been found more attracted by the middle or upper part of all the plants (personal observation by PH and YCD). Among various benefits, omnivorous Heteropterans may have to get water through plant feeding activities to produce the saliva needed for the extra‐oral prey digestion (Cohen 1998; Sinia et al. 2004). It is also possible that the simultaneous decrease in plant and prey feeding by *M. pygmaeus* may be attributed to increased concentrations of secondary compounds in plants, such as toxins and other defensive compounds, when the tomato plants suffered water limitation (Han et al. submitted data). Inbar et al. (2001) reported significant increases in many defensive compounds such as chitinase, glucanase, peroxidase activities as well as concentrations of phenolics in water‐stressed tomato plants. Among these compounds, peroxidase has an impact on food digestibility and protein availability to herbivores (Duffey and Stout 1996), and phenolics, especially rutin and chlorogenic acid, were considered to affect the performance of herbivorous insects on tomato plants (Wilkens et al. 1996; English‐Loeb et al. 1997; Inbar et al. 2001). Hence, we assumed that the enhanced defensive traits in water‐stressed tomato plants may be another factor resulting in decreased plant feeding by *M. pygmaeus*, even though it was still unclear what those defensive compounds are (Kaplan and Thaler 2011). However, our data did not show the effects of nitrogen inputs in determining the feeding decisions between plant and/or prey source by *M. pygmaeus*. This does not necessarily mean that this factor could be ruled out when exploring the mechanisms governing the specific feeding strategy adopted by a given omnivorous species.

The low water input to plants significantly reduced *M. pygmaeus* longevity on plants bearing prey (Table 2, Fig. 5). Since omnivorous predators need water for prey digestion (Cohen 1995), water limitation may disturb physiological processes involved in such prey digestion and may lead to decreased fitness and/or longevity. In addition, the significant interaction between prey availability and water input might suggest that prey feeding increased water stress in *M. pygmaeus*, which has been also reported on another species by Sinia et al. (2004). Instead of being affected by nitrogen and water treatments, *M. pygmaeus* fertility was positively enhanced with the presence of prey (Fig. 5), which allows for full reproductive achievement (Vandekerkhove and De Clercq 2010). Other predaceous bugs also need prey as food sources to achieve optimal reproduction rates (Richards and Schmidt 1996; Cocuzza et al. 1997). However, we lacked the assessment of the possible treatment impact (water and/or nitrogen) on actual prey food quality in our current study since factitious prey was used. The nutritive values of prey eggs produced by individuals that developed on tomato plants may vary due to different nitrogen and water treatments, and this variation may indirectly affect the omnivorous predator. For example, *M. pygmaeus*, like other omnivorous Heteropterans, has been increasingly reported attacking *Tuta absoluta*, a major pest on tomato plants, alone or together with other natural enemies in the Afro‐Eurasian continent (Chailleux et al. 2013a,b; Zappalà et al. 2013) and we have recently demonstrated that the pest can be affected through bottom‐up effects when modulating water and nitrogen inputs (Han et al. 2014). It is unclear whether these effects on *T. absoluta*, and/or other prey, may lower the nutritive quality of eggs attacked by the predators, provoking a cascade impact on the *M. pygmaeus* population dynamics. In our biological model, however, this may not be a major factor since *T. absoluta* represents actually a poor‐quality food for *M. pygmaeus* when it is consumed as a single prey (Jaworski et al. 2013; Mollá et al. 2014), although the predator is known to consume this specific prey on tomato plants.

Our current data were not able to provide robust evidence to support any of the three hypotheses: *facilitation*,* switching*, or *independence* described earlier. However, the negative impact of water limitation in plants on *M. pygmaeus* may indirectly indicate that they need to acquire water by feeding on plant tissue(s) to assist prey predation and/or digestion. In practice, the efficacy of *M. pygmaeus* in biocontrol programs may be optimized by the sufficient water inputs to the crops. Nonetheless, the need of plant feeding may potentially cause injury to plants, especially under the conditions with high predator densities and low prey availability (Castañé et al. 2011). In this context, plants face a trade‐off between costs (increase in injuries caused by predators) and benefits (decrease in damage caused by the herbivorous pest).

In conclusion, our case study with the omnivorous predator *M. pygmaeus* demonstrated that plant water status may be a limiting factor for the foraging behavior and fitness of the omnivorous predator. Our study provides insights for further studies on the feeding ecology of omnivorous predators. The ecological importance of phytophagous behavior in omnivorous predator may be quite important as it could vary in response to plant quality changes. The degree of omnivory can actually largely affect strength of top‐down forces on various pests. This may result in trophic cascades, altering the stability of food webs with unexpected influence on transfer of nutrients across arthropod communities (Polis and Strong 1996; Holt and Polis 1997; Thompson et al. 2007). The present work highlights the need of better knowledge on the role of phytophagous behavior exhibited by omnivorous arthropods, and on how it may shape the structure of arthropod communities in natural or managed ecosystems.

## Conflict of Interest

The authors declare that they have no conflict of interest.
